# Biphasic Adaptations of Gastric Epithelial Cells in Chronic *H. pylori* Infection from Stress to Tolerance

**DOI:** 10.3390/ijms26189016

**Published:** 2025-09-16

**Authors:** Xiulin Zhang, Yang He, Xiaolu Zhang, Ziyi Liang, Wendong Wang, Zhenyu Da, Jianyi Lv, Meng Guo, Xueyun Huo, Xin Liu, Jing Lu, Lixue Cao, Xiaoyan Du, Zhongming Ge, Zhenwen Chen, Xuancheng Lu, Jianzhong Zhang, Changlong Li

**Affiliations:** 1 Beijing Key Laboratory of Cancer Invasion and Metastasis Research, Department of Medical Genetics and Developmental Biology, School of Basic Medical Science, Capital Medical University, Beijing 100069, China; xiulinzh@163.com (X.Z.);; 2 School of Nursing, Dalian Medical University, Dalian 116044, China; heyang_hello@163.com; 3 Department of Molecular Cell Biology, Graduate School of Medical Sciences, Kyushu University, Higashi-ku, Fukuoka 812-8582, Japan; 4 Division of Comparative Medicine, Massachusetts Institute of Technology, Cambridge, MA 02139, USA; 5 National Key Laboratory of Intelligent Tracking and Forecasting for Infectious Diseases, Chinese Center for Disease Control and Prevention (Chinese Academy of Preventive Medicine), Beijing 102206, China; 6 National Key Laboratory of Intelligent Tracking and Forecasting for Infectious Diseases, National Institute for Communicable Disease Control and Prevention, Chinese Center for Disease Control and Prevention (Chinese Academy of Preventive Medicine), Beijing 102206, China

**Keywords:** *Helicobacter pylori*, chronic infection, gastric epithelial cells

## Abstract

*Helicobacter pylori* (*H. pylori*) is a well-known pathogen associated with chronic gastric infection, progressing from gastritis to gastric adenocarcinoma, but the dynamic phenotypic and molecular characteristics of gastric epithelial cells during sustained infection remain unclear. We established a chronic infection model using the human gastric epithelial cell line GES-1, exposed to *H. pylori* or its lysate across 30 generations, dynamically assessing cell proliferation, migration, invasion, apoptosis, autophagy, and epithelial–mesenchymal transition (EMT) markers, with RNA sequencing for transcriptomic changes and a Mongolian gerbil model to validate chronic pathological progression. Acute *H. pylori* exposure induced pronounced morphological changes; suppressed proliferation, migration, and invasion; triggered apoptosis; and blocked autophagic flux, while long-term stimulation reversed these effects. EMT markers showed progressive loss of epithelial characteristics with chronic infection. RNA sequencing revealed a dynamic shift from inflammation-driven apoptosis to adaptive survival mechanisms. In vivo, prolonged infection induced dynamic TLR expression alongside progressive gastric pathology, including atrophy and dysplasia. Our study provides new molecular evidence for dynamic cellular and immunological adaptations of gastric epithelial cells under chronic *H. pylori* infection, highlighting critical intervention windows for preventing gastric carcinogenesis.

## 1. Introduction

*Helicobacter pylori* (*H. pylori*) is a microaerophilic Gram-negative bacterium that colonizes the human gastric mucosa. Classified as a Group I carcinogen by the World Health Organization (WHO), it plays a critical role in gastric carcinogenesis—a condition that has remained a significant global health burden for decades [1,2,3]. Chronic infection with *H. pylori* is considered an evidential risk factor for non-cardia gastric cancer development in several cohort studies and associated with the progression from gastritis to atrophic gastritis, intestinal metaplasia, dysplasia, and, ultimately, gastric adenocarcinoma [4,5]. Numerous studies have highlighted the role of *H. pylori* in disrupting gastric epithelial homeostasis by delivering virulence factors such as cytotoxin-associated gene A (CagA) into host cells through its Type IV Secretion System (T4SS) [6,7,8]. These bacterial components perturb host signaling pathways, thereby influencing key cellular processes: proliferation, apoptosis, autophagy, and epithelial–mesenchymal transition (EMT) [9,10]. Toxic substances from *H. pylori*’s long-term metabolic lysis contribute to gastric epithelial carcinogenesis; these lysates, containing key virulence factors, recapitulate pathogen–mucosa interactions and drive host responses.

Notably, while these mechanistic insights have advanced our understanding, the experimental models used to investigate such processes require critical evaluation. Although previous studies have frequently utilized acute exposure models to investigate *H. pylori*-induced pathogenesis, the infection is inherently chronic in vivo, persisting for years due to *H. pylori*’s intrinsic colonization capacity [11,12]. Consequently, short-term in vitro models may inadequately recapitulate the sustained interactions between *H. pylori* and gastric epithelial cells. Several studies have successfully established chronic *H. pylori* infection cell models, which demonstrate altered cell morphology and EMT properties that promote the malignant transformation of gastric epithelial cells [12,13,14]. However, the dynamic functional and inflammatory changes and characteristics of gastric epithelial cells during prolonged *H. pylori* stimulation remain insufficiently characterized.

In this study, we aimed to characterize the dynamic biological behavior of gastric epithelial cells during long-term *H. pylori* infection. Using the human gastric epithelial cell line GES-1 as an in vitro model, we investigated how sustained exposure to *H. pylori* and its components modulates cell proliferation, migration, and invasion as well as the EMT process—along with the corresponding regulatory patterns of gene expression and associated signaling pathways. Our findings provide novel insights into the cellular dynamics underlying *H. pylori*-associated gastric epithelial transformation and facilitate a more comprehensive understanding of gastric cancer initiation.

## 2. Results and Discussion

### 2.1. GES-1 Cells Undergo Morphological Reversion, CagA Fluctuation, and Immune Desensitization During H. pylori Infection

To investigate the dynamic morphological and infectious responses of gastric epithelial cells to *H. pylori* over time, GES-1 cells were chronically co-cultured with *H. pylori* for 30 generations. We then analyzed cells subjected to either acute (GES-1 *Hp* F1) or sustained (GES-1 *Hp* F30) bacterial exposure.

**Figure 1:**
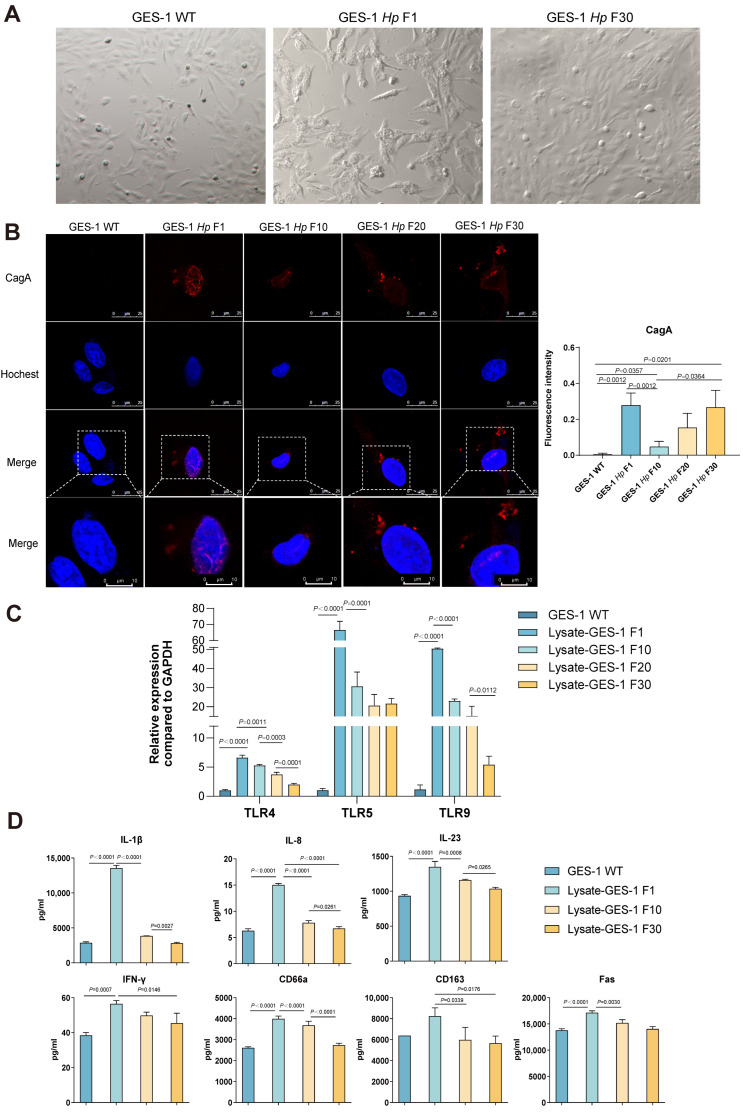
The morphology and infectious characteristics of GES-1 cells under *H. pylori* infection. (**A**) The morphology of normal gastric epithelial cells (GES-1 WT) and *H. pylori* short-term (GES-1 *Hp* F1) or long-term (GES-1 *Hp* F30) infected gastric epithelial cells. Magnification: 1000×. (**B**) IF staining of CagA delivered to GES-1 cells as *H. pylori* infection prolonged. Red represents CagA protein, blue represents the nucleus. (**C**) The mRNA level of TLR4, TLR5, and TLR9 in GES-1 WT cells and *H. pylori* lysate-stimulated GES-1 cells (Lysate-GES-1 F1, Lysate-GES-1 F10, Lysate-GES-1 F20, Lysate-GES-1 F30). (**D**) The expression of pro-inflammatory cytokines (IL-1β, IL-8, IL-23, IFN-γ) and molecules (CD66a, CD163, Fas) in GES-1 WT, Lysate-GES-1 F1, Lysate-GES-1 F10, and Lysate-GES-1 F30 cells detected by multiplex assay. All experiments were performed with 3 biological replicates. One-way ANOVA was used to determine statistical significance in (**B**–**D**).

Upon short-term *H. pylori* stimulation, GES-1 *Hp* F1 cells exhibited a characteristic hummingbird phenotype, marked by elongated morphology—consistent with CagA-mediated cytoskeletal rearrangements reported in previous studies [12]. This acute morphological alteration, however, gradually diminished with prolonged exposure. By the 30th generation (GES-1 *Hp* F30), infected cells had reverted to morphologies similar to those of untreated cells (Figure 1A).

IF analysis of CagA revealed that, in GES-1 *Hp* F1 cells, CagA signals were highly enriched in the cytoplasm. By the 10th generation, CagA fluorescence intensity decreased significantly. Notably, with further progression to the 30th generation (GES-1 *Hp* F30), CagA signals rebounded, indicating the development of immune tolerance with attenuated clearance of bacterial toxins (Figure 1B). During *H. pylori* gastric colonization, besides T4SS-mediated CagA delivery exerting cytotoxic effects, bacterially released toxic proteins continuously stimulate gastric epithelial cells.

In infected individuals, gastric epithelial Toll-like receptors (TLRs) serve as early immune sensors for bacterial components. Toxic substances released during *H. pylori* proliferation and lysis are recognized by TLRs; such lysates act as pathogenic facilitators in chronic *H. pylori* infection. We established a GES-1 co-culture model with *H. pylori* lysate to mimic persistent colonization; quantified TLR4, TLR5, and TLR9 RNA levels in cells at passages 1, 10, 20, and 30 post-lysate stimulation; and concurrently measured inflammatory factors and cytokines in culture supernatants. Initial lysate stimulation (Lysate-GES-1 F1) significantly upregulated TLR4, TLR5, and TLR9 mRNA in GES-1 cells, with gradual downregulation during prolonged exposure (Figure 1C). This aligned with progressive immune desensitization, as multiplex assays showed peak secretion of IL-1β, IL-8, IL-23, IFN-γ, CD66a, CD163, and Fas after first-generation *H. pylori* lysate stimulation, followed by gradual decline with extended treatment (Figure 1D).

While *H. pylori* pathogenicity and models are well-characterized [12,13,14,15,16,17,18], few studies address dynamic epithelial changes during inflammation and malignant transformation. Our 30-passage chronic model fills this gap by identifying key adaptive processes in infected GES-1 cells. Acute infection induces the “hummingbird” phenotype [19,20], but prolonged exposure drives morphological reversion—reflecting cellular adaptation to bacterial stress. This aligns with CagA dynamics: acute enrichment, mid-term F10 reduction via initial immune clearance, and late F30 rebound, matching our prior immune tolerance report and indicating attenuated toxin clearance in persistent colonization [13].

*H. pylori* lysates trigger TLR-mediated inflammatory adaptation [21]. Initial stimulation upregulates TLR4/5/9, which are critical for bacterial recognition, and peaks pro-inflammatory cytokine and immune molecule secretion [22,23]. Prolonged exposure reduces TLR expression and inflammation, revealing desensitization that balances tissue damage and colonization.

Collectively, morphological reversion, CagA rebound, and TLR desensitization form an adaptive state facilitating long-term *H. pylori* colonization, advancing understanding of host-cell adaptation in chronic infection and gastric disease progression.

### 2.2. GES-1 Cell Proliferation Is Impaired in Acute H. pylori Infection and Gradually Adapts upon Sustained Exposure

**Figure 2:**
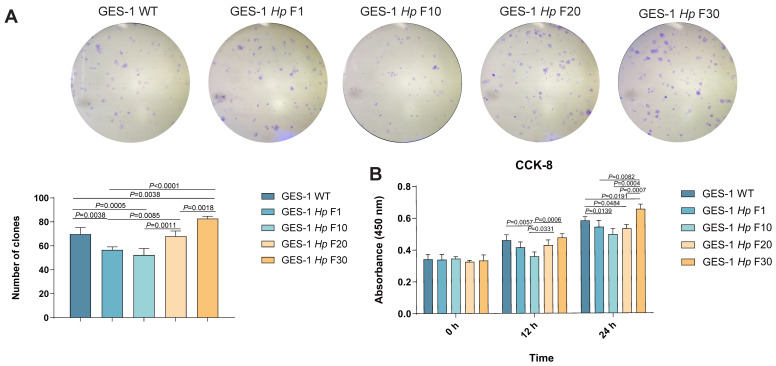
The proliferation capacity of GES-1 cells under *H. pylori* infection. (**A**) The proliferation capacity of GES-1 WT, GES-1 *Hp* F1, GES-1 *Hp* F10, GES-1 *Hp* F20, and GES-1 *Hp* F30 cells analyzed by colony formation assay. (**B**) The proliferation capacity of GES-1 WT, GES-1 *Hp* F1, GES-1 *Hp* F10, GES-1 *Hp* F20, and GES-1 *Hp* F30 cells analyzed by CCK-8 cell proliferation assay. All experiments were performed with 3 biological replicates. One-way ANOVA was used to determine statistical significance in (**A**). Two-way ANOVA was used to determine statistical significance in (**B**).

To evaluate the impact of *H. pylori* infection on gastric epithelial cell proliferation, we analyzed the proliferation capacity of GES-1 WT, GES-1 *Hp* F1, GES-1 *Hp* F10, GES-1 *Hp* F20, and GES-1 *Hp* F30 cells with colony formation and CCK-8 assays. In the early phase of infection (GES-1 *Hp* F1 and F10), GES-1 cells displayed a marked decrease in proliferation capacity compared with the untreated control group. Colony numbers were significantly reduced, and the CCK-8 assay also indicated suppressed cell viability, suggesting an acute stress response to initial bacterial exposure. However, as co-culture with *H. pylori* was extended over multiple passages, a gradual recovery in proliferation capacity was observed in GES-1 *Hp* F20 and GES-1 *Hp* F30 cells (Figure 2A,B).

These findings indicate that acute *H. pylori* exposure severely impairs GES-1 proliferation, whereas long-term infection drives gradual adaptive responses that mitigate this impairment.

### 2.3. Migration and Invasion Capacity of GES-1 Decreased in Acute H. pylori Infection, with Gradual Adaptation Under Sustained Exposure

To clarify *H. pylori*’s effect on gastric epithelial motility, we assessed migration and invasion across infection stages via wound-healing and transwell assays.

**Figure 3:**
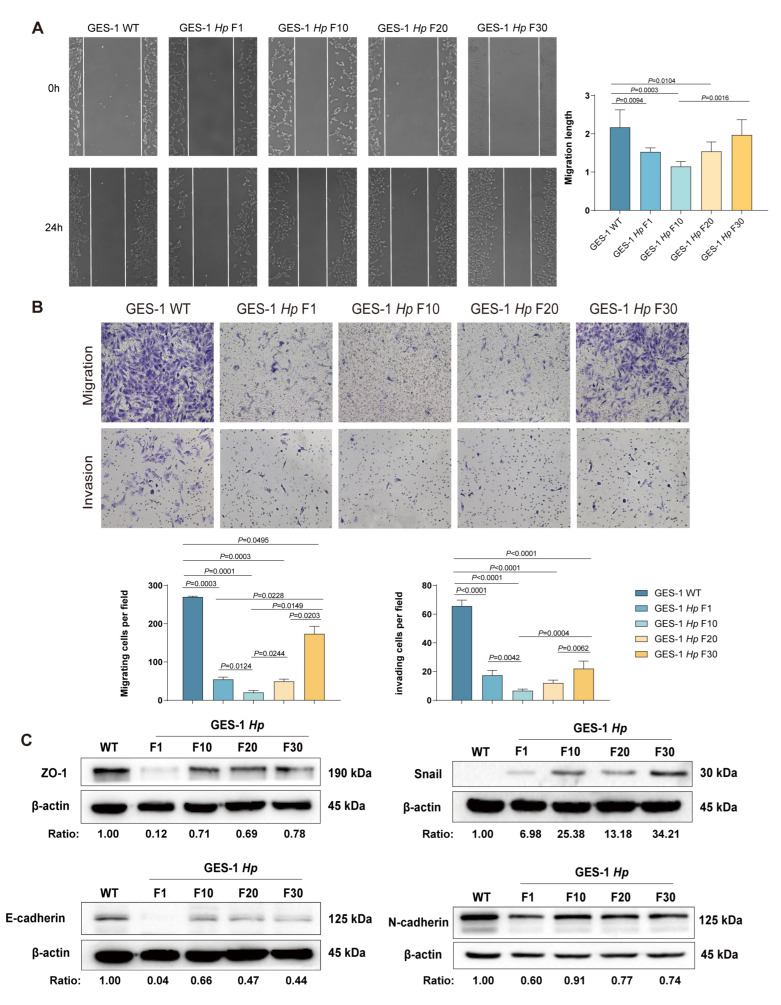
The migration, invasion capacity, and EMT features of GES-1 cells under *H. pylori* infection. (**A**) The proliferation capacity of GES-1 WT, GES-1 *Hp* F1, GES-1 *Hp* F10, GES-1 *Hp* F20, and GES-1 *Hp* F30 cells analyzed by colony formation assay. (**B**) The proliferation capacity of GES-1 WT, GES-1 *Hp* F1, GES-1 *Hp* F10, GES-1 *Hp* F20, and GES-1 *Hp* F30 cells analyzed by CCK-8 cell proliferation assay. (**C**) The expression of EMT markers (ZO-1, E-cadherin, Snail and N-cadherin) detected by Western blotting. All experiments were performed with 3 biological replicates. One-way ANOVA was used to determine statistical significance in (**A**,**B**).

Following acute *H. pylori* exposure (GES-1 Hp F1), GES-1 cells exhibited a significant reduction in migration distance and transwell migration rate compared to uninfected controls (GES-1 WT), with invasion ability also significantly suppressed (Figure 3A,B), indicating early infection compromises cytoskeletal remodeling and cell viability. Interestingly, as infection was prolonged over several generations, GES-1 Hp F30 cells showed recovered wound closure and increased transwell movement compared with GES-1 Hp F1, F10, and F20 (Figure 3A,B).

We further evaluated EMT features in infected GES-1 cells for mechanisms. As shown in Figure 3C, epithelial markers E-cadherin and ZO-1 were downregulated, while the mesenchymal marker Snail was upregulated in GES-1 Hp F30 compared to GES-1 WT cells.

Decreased expression of E-cadherin with increased Snail in GES-1 *Hp* F30 cells also demonstrated a trend of malignant transformation and cancer initiation after long-term infection [24,25]. These results reveal a biphasic response in which initial bacterial stress dampens cell motility, while chronic infection reprograms the cellular phenotype to a more motile and invasive state, a transformation that has the potential to facilitate malignant progression.

### 2.4. Acute H. pylori Infection Disrupts Autophagy and Apoptosis in GES-1 Cells, with Adaptation Restoring Homeostasis in Chronic Stages

**Figure 4:**
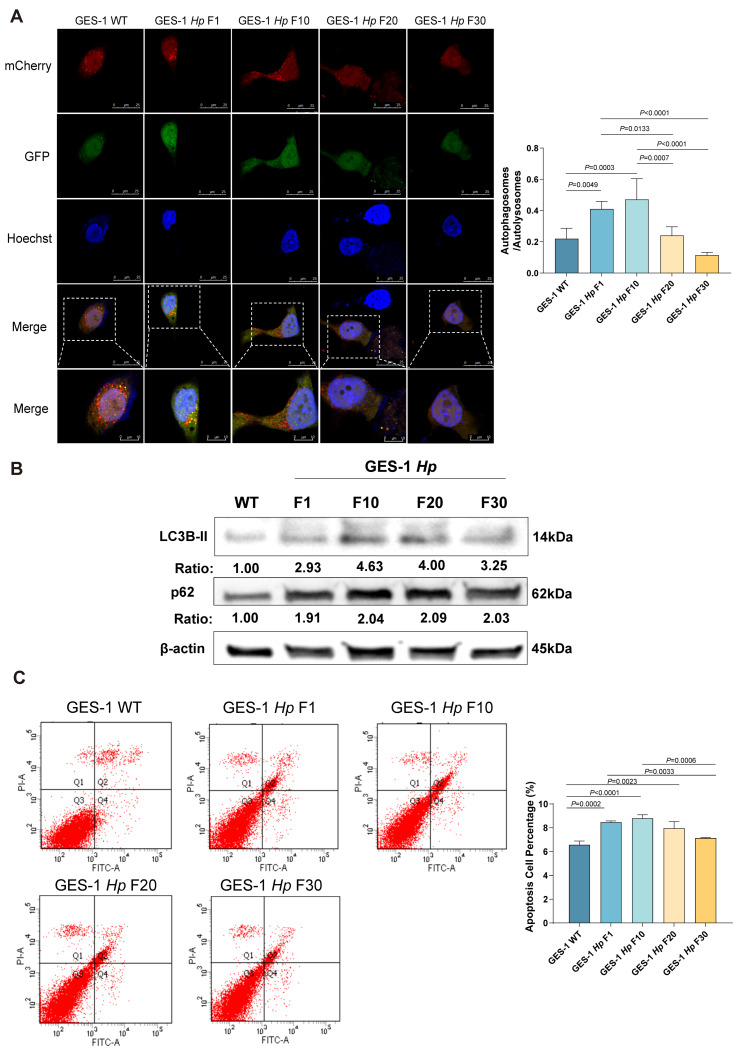
The autophagy and apoptosis of GES-1 cells under *H. pylori* infection. (**A**) The fluorescence image of autophagosomes and autolysosomes and the ratio of autophagosome counts/autolysosome counts in GES-1 WT, GES-1 *Hp* F1, GES-1 *Hp* F10, GES-1 *Hp* F20, and GES-1 *Hp* F30 cells (calculated with 5 cells for each group). (**B**) The expression of autophagy markers (LC3-II and p62) detected by Western blotting (performed in triplicate). (**C**) The apoptosis level of GES-1 WT, GES-1 *Hp* F1, GES-1 *Hp* F10, GES-1 *Hp* F20, and GES-1 *Hp* F30 cells detected by flow cytometry (performed in triplicate). All analyses used one-way ANOVA with Tukey’s test.

To investigate the dynamic regulation of autophagy in gastric epithelial cells during different stages of *H. pylori* infection, GES-1 cells were transfected with an EGFP-mCherry-LC3 lentivirus and monitored across multiple infection generations, with the number of yellow (representing autophagosomes) and red puncta (representing autolysosomes) indicated by LC3. In early-stage infection (GES-1 *Hp* F1), confocal imaging revealed both a quantitative and proportional increase in autophagosomes, as the ratio of autophagosome/autolysosome counts has elevated (0.41 ± 0.05) when compared with the uninfected cells (0.22 ± 0.07), indicating a blockage in autophagic flux. This inhibition was further aggravated in GES-1 *Hp* F10 cells, where the ratio of autophagosomes to autolysosomes significantly increased (0.47 ± 0.13), suggesting impaired fusion of autophagosome to lysosome. By the 20th and 30th generations of GES-1 cells post-infection (GES-1 *Hp* F20 and GES-1 *Hp* F30), the autophagic flux had largely recovered, with the distribution and quantity of puncta comparable to those of uninfected control cells (0.24 ± 0.06 for GES-1 *Hp* F20, 0.11 ± 0.02 for GES-1 *Hp* F30) (Figure 4A). Western blot analysis of LC3-II and p62 confirmed that, compared to controls, GES-1 *Hp* F1 cells showed increased LC3-II and p62 expression, with further elevation in GES-1 *Hp* F10. Conversely, GES-1 Hp F20 and F30 cells exhibited reduced LC3-II and p62 levels, confirming the restoration of autophagic flux in the late stage of infection (Figure 4B).

Apoptotic analysis by flow cytometry showed a transient elevation in apoptosis rates in GES-1 *Hp* F1 (8.47% ± 0.12%) and GES-1 *Hp* F10 cells (8.80% ± 0.30%). However, apoptosis levels significantly declined in GES-1 *Hp* F30 cells (7.13% ± 0.06%), returning to levels that were almost the same as those in untreated control cells (GES-1 WT) (6.57% ± 0.32%) (Figure 4C).

Taken together, these findings indicate that, while acute *H. pylori* infection disrupts both autophagy and apoptosis in GES-1 cells, long-term bacterial stimulation leads to cellular adaptation and tolerance against the infection. The restoration of autophagic flux and suppression of apoptosis at later stages may contribute to enhanced survival of gastric epithelial cells under chronic *H. pylori* colonization.

For the functional features of *H. pylori*-infected gastric epithelial cells, our study revealed how chronic infection reshapes cell fate. Autophagy blockage and increased apoptosis during the acute infection represent host defense mechanisms against stress, and this might be attributed to CagA-mediated signaling according to previous studies [26,27]. However, these processes are reversed under long-term infection, as restored autophagic flux provides a survival advantage, while inhibited apoptosis reduces cell death [28,29].

### 2.5. Bulk RNA-Seq Reveals Inflammation Reprogramming in GES-1 Cells During Sustained H. pylori Lysate Stimulation

**Figure 5:**
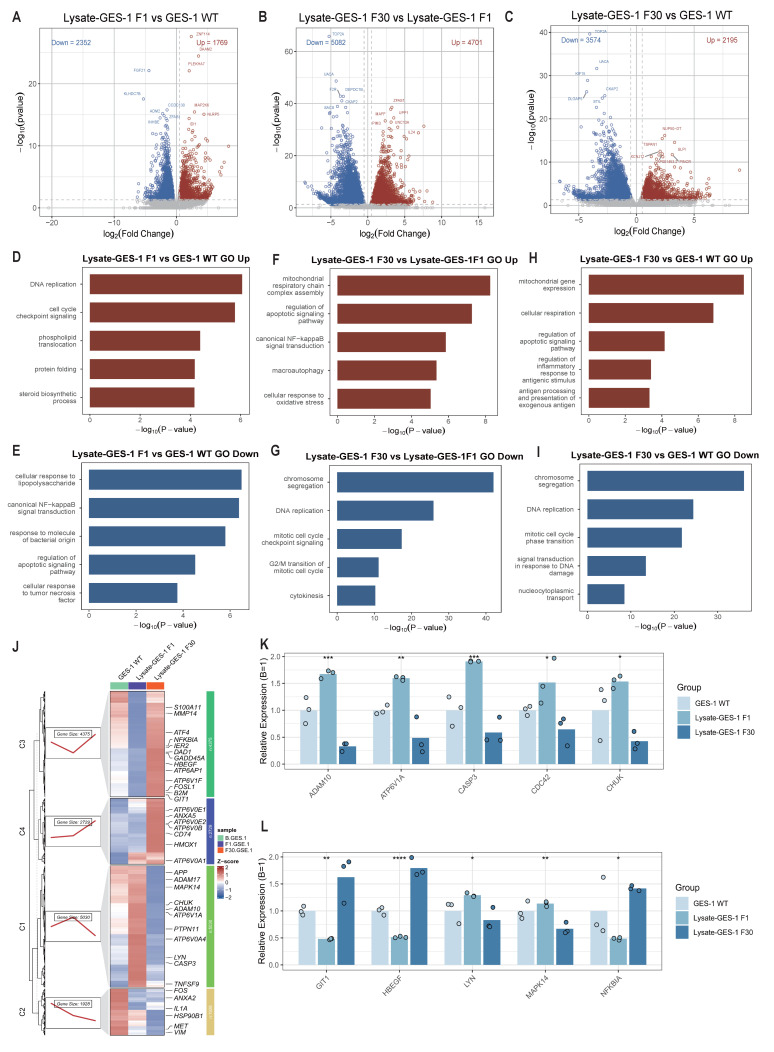
Transcriptomic remodeling of gastric epithelial cells in response to incremental *H. pylori* lysate stimulation. (**A**–**C**) Volcano plots for Lysate-GES-1 F1 vs. GES-1 WT, Lysate-GES-1 F30 vs. GES-1 WT, and Lysate-GES-1 F30 vs. Lysate-GES-1 F1. Red dots indicate genes upregulated in the first-named group, and blue dots indicate downregulated genes (|log_2_FC| ≥ 1, *p* < 0.05). Numbers in the plot corners denote the counts of up- and downregulated genes. (**D**–**I**) Gene Ontology enrichment generated with clusterProfiler, ranked by −log_10_(*p*); red bars represent pathways enriched for upregulated genes, and blue bars represent pathways enriched for downregulated genes. (**J**) Unsupervised TCseq clustering heatmap showing DEGs with dynamic expression patterns across infection stages; representative gene names are annotated on the right. (**K**,**L**) Relative expression of selected core genes (normalized to GES-1 WT = 1). Points represent biological replicates (*n* = 3). One-way ANOVA was used to determine statistical significance in (**K**,**L**). (* *p* < 0.05, ** *p* < 0.01, *** *p* < 0.001, **** *p* < 0.0001).

Bulk RNA sequencing was performed on gastric epithelial cells from untreated controls (GES-1 WT) and groups stimulated with *H. pylori* lysate for 1 (Lysate-GES-1 F1) or 30 generations (Lysate-GES-1 F30). The RNA-seq data have been uploaded to the NCBI database (GSE149092). Compared to untreated cells, Lysate-GES-1 F1 showed 4121 differentially expressed genes, while 9783 DEGs were identified in Lysate-GES-1 F30 versus Lysate-GES-1 F1, reflecting intensified transcriptional responses with prolonged lysate exposure (Figure 5A–C, Supplementary File S1).

Gene Ontology (GO) enrichment analysis revealed upregulated “DNA replication” and “cell-cycle checkpoint” pathways in Lysate-GES-1 F1, indicating activated self-repair mechanisms following acute stimulation by *H. pylori* components. In contrast, chronic stimulation (Lysate-GES-1 F30) activated pathways including “mitochondrial gene expression” and “canonical NF-κB signal transduction”, reflecting enhanced cellular metabolism and potential carcinogenic activation (Figure 5D–I, Supplementary File S2). Sustained *H. pylori* lysate stimulation induced significant transcriptional reprogramming in GES-1 cells. Acute exposure (F1) triggered DNA repair and cell-cycle checkpoint pathways, reflecting an immediate compensatory response. In contrast, chronic stimulation (F30) provoked more profound transcriptomic alterations, prominently activating mitochondrial gene expression and NF-κB signaling. These changes suggest a metabolic reorganization and sustained inflammatory activation, consistent with features of premalignant transformation driven by chronic inflammation. Our findings imply that long-term *H. pylori* infection may facilitate gastric carcinogenesis through NF-κB-mediated inflammatory microenvironment.

Unsupervised clustering further showed that genes in the inflammation-apoptosis C1 module were upregulated in Lysate-GES-1 F1 but downregulated in Lysate-GES-1 F30, while stress-protective C3 module genes were markedly downregulated in Lysate-GES-1 F1 and recovered after 30 generations of stimulation (Figure 5J). Notably, MMP14, IER2, HBEGF, and GIT1 were highly expressed in Lysate-GES-1 F30, indicating enhanced epithelial motility. Bar plots confirmed that sustained stimulation significantly repressed apoptosis-stress-related genes while activating those involved in migration and proliferation (Figure 5K,L).

Collectively, sustained *H. pylori* lysate exposure first triggers energetic compensation, followed by coordinated inflammation-cell cycle reprogramming, offering a metabolic–immunologic framework for understanding chronic gastric mucosal injury and pre-neoplastic progression.

Results from bulk RNA-seq analyses are also consistent with the dynamic phenotypes of autophagy and apoptosis, in which genes of inflammation and apoptosis were activated under acute stimulation of *H. pylori* lysate stimulation, with enhanced stress-protective capacity after chronic treatment.

### 2.6. Chronic H. pylori Infection Induces Progressive Gastric Pathology and Immune Tolerance in Mongolian gerbils

**Figure 6:**
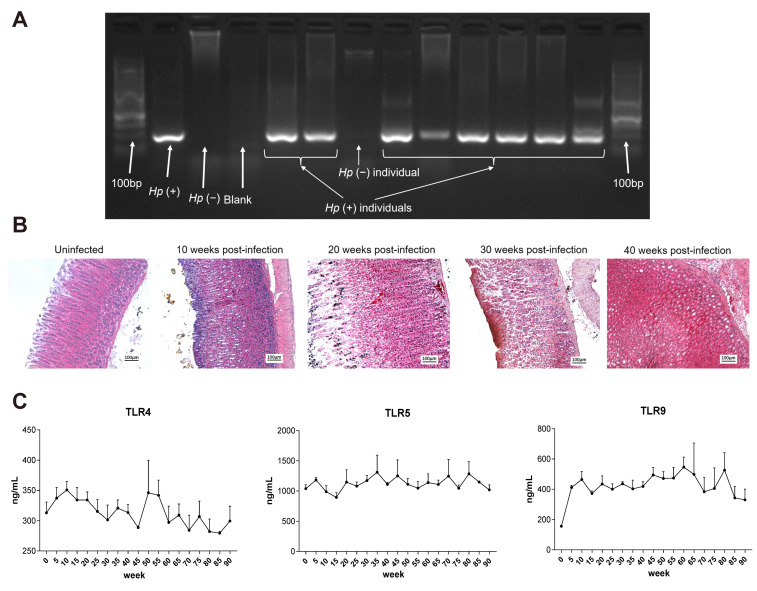
The progressive pathologic changes in the gastric tissues of *Mongolian gerbils*. (**A**) The colonization of *H. pylori* detected by nested PCR with gastric fluid from infected *Mongolian gerbils*. (**B**) The representative pathologic changes in gastric tissue from *Mongolian gerbils* during long-term *H. pylori* infection. (**C**) The expression of TLR4, TLR5, and TLR9 in the serum of *H. pylori*-infected *Mongolian gerbils*.

To investigate the long-term in vivo effects of *H. pylori* infection on gastric tissue, we established a chronic infection model in *Mongolian gerbils* using *H. pylori* strain 7.13, a gerbil-adapted strain known to induce gastric carcinogenesis in this species. After 10 weeks of infection, gastric juice was collected and subjected to nested PCR to confirm successful bacterial colonization (Figure 6A), with only *H. pylori*-positive animals included in subsequent analyses.

Histopathological assessment revealed progressive structural alterations in the gastric mucosa of gerbils infected with *H. pylori* strain 7.13 as the infection persisted. While no pathological changes were observed at 10 weeks post-infection, various lesions emerged with prolonged infection: glandular atrophy was detected in *H. pylori*-colonized gerbils at 20 and 30 weeks post-infection, and dysplasia appeared in the gastric tissue by 40 weeks post-infection (Figure 6B). These pathological changes closely mimic the cascade of gastric carcinogenesis observed in human *H. pylori*-related disease.

To further evaluate host immune responses during prolonged infection, we quantified serum expression levels of Toll-like receptors (TLR4, TLR5, and TLR9) in *Mongolian gerbils* infected with *H. pylori* strain ATCC 43504 for 90 weeks. Notably, both TLR4 and TLR9 levels increased during early infection stages but declined in later phases of the extended infection model (Figure 6C), indicating initial activation of the innate immune response followed by immunologic adaptation or tolerance as the infection became chronic.

These findings align with our previous observations that long-term *H. pylori* infection induces host immune desensitization, which facilitates bacterial persistence and contributes to pathological progression.

The in vivo experiments with *H. pylori* strain 7.13 exhibited a process of *H. pylori*-induced gastric pathogenesis that followed Correa’s cascade: normal (10 weeks post-infection), atrophic gastritis (20 to 30 weeks post-infection), dysplasia (40 weeks post-infection). This result demonstrated a time-dependent pathological progression that further validated our cellular-level findings and provided significant insights into the clinical identification of high-risk patients and optimal intervention timing.

## 3. Material and Methods

### 3.1. Bacteria Strain and Bacteria Culture

*H. pylori* strain ATCC 43504 (CagA^+^, VacA^+^) was provided by the National Institutes for Food and Drug Control, Beijing, China. *H. pylori* strain 7.13 (CagA^+^, VacA^+^) was kindly provided by Dr. Nonghua Lv from the Department of Gastroenterology, First Affiliated Hospital of Nanchang University, Nanchang, China. Bacteria were cultured on Karmali agar plates (OXOID, Waltham, MA, USA, CM0935) supplemented with 5% sterile and defibrinated sheep blood (MRC, Cambridge, UK, CCS30037.01) at 37 °C under the condition of 5% O_2_, 10% CO_2_, and 85% N_2_.

### 3.2. Cell Lines and Cell Treatment

Human gastric epithelial cell line GES-1 was purchased from Ningbo Mingzhoubio Biotechnology Co., Ltd. (Ningbo, China) Cells were cultured in DMEM (VISTECH, Hartford, CT, USA, VM-1101BM) supplemented with 10% fetal bovine serum (FBS) (VISTECH, SE100-011) and 1% penicillin/streptomycin antibiotics solution (KeyGen BioTECH, Nanjing, China, KGY0023).

GES-1 cells for *H. pylori* infection were cultured in antibiotic-free medium with *H. pylori* strain ATCC 43504 (CagA^+^, VacA^+^) at a multiplicity of infection (MOI) of 1000:1 at 37 °C under 5% CO_2_ condition for 24 h, then washed 5 times with phosphate-buffered saline (PBS) (ROBY, Beijing, China, RBR105S-500) to remove *H. pylori*, and cultured in bacteria-free medium at 37 °C under 5% CO_2_ for another 24 h as one generation (GES-1 *Hp* F1). Cells co-cultured with *H. pylori* for 10, 20, and 30 consecutive generations were marked as GES-1 *Hp* F10, GES-1 *Hp* F20, and GES-1 *Hp* F30, respectively. GES-1 cells without *H. pylori* infection were regarded as the negative control and marked as GES-1 WT.

The *H. pylori* strain ATCC 43504 suspension was placed on ice and sonicated at 100 W power for 30 s, repeated 10 times with 20-s intervals between each sonication cycle. The resulting suspension was centrifuged at 12,000× *g* for 10 min, and the supernatant was collected. Protein concentration was determined using a BCA protein quantitation kit (Thermo, Waltham, MA, USA), after which the supernatant was diluted with PBS to a final concentration of 200 μg/mL to prepare the *H. pylori* lysate.

GES-1 cells for *H. pylori* lysate treatment were cultured in medium containing lysate generated from *H. pylori* strain ATCC 43504 (CagA^+^, VacA^+^) at a final concentration of 2 μg/mL at 37 °C under 5% CO_2_ condition for 24 h, then washed once with PBS to remove the medium with *H. pylori* lysate, and cultured in normal medium for another 24 h as one generation (Lysate-GES-1 F1). Cells co-cultured with *H. pylori* lysate for 30 consecutive generations were marked as Lysate-GES-1 F30.

### 3.3. Animal Infection

All *Mongolian gerbils* used for *H. pylori* infection were 6–8 weeks old, with a body weight of 50–60 g. Gerbils without *H. pylori* infection served as the uninfected control group. For the 40-week infection experiment using *H. pylori* strain 7.13, gerbils were infected for 10–40 weeks and analyzed at consecutive time points (0, 10, 20, 30, and 40 weeks post-infection). In the 90-week infection experiment with *H. pylori* strain ATCC 43504 (CagA^+^, VacA^+^), gerbils were infected for 5–90 weeks and analyzed at consecutive time points (every 5 weeks from 0 to 90 weeks post-infection). The animal study was reviewed and approved by the Animal Experiments and Experimental Animal Welfare Committee of CMU (protocol: AEEI-2015-179; 20 September 2018). All procedures were conducted in accordance with the institutional Guidelines for the Care and Use of Laboratory Animals.

### 3.4. Immunofluorescence

Cells and stomach tissue sections were incubated overnight at 4 °C with mouse anti-CagA antibody (Santa Cruz, Dallas, TX, USA, sc-28368, 1:200 dilution for immunofluorescence (IF)), followed by incubation with Alexa Fluor^®^ 555-conjugated goat anti-mouse IgG (HUABIO, Hangzhou, China, HA1117, 1:200 dilution). The cell nuclei were counterstained with Hoechst33342 (Solarbio, Beijing, China, C0021, 10 μg/mL).

### 3.5. CCK-8 Cell Proliferation Assay

The cell proliferation capacity was analyzed using Cell Counting Kit-8 (CCK-8) solution (Vazyme, Nanjing, China, A311-01). GES-1 WT, GES-1 *Hp* F10, GES-1 *Hp* F20, and GES-1 *Hp* F30 cells were counted and seeded into a 96-well plate at a density of 3 × 10^3^ cells/well. At 0 h, 12 h, and 24 h time points post-cell attachment, 10% CCK-8 solution was added to each well, and cells were incubated at 37 °C under 5% CO_2_ condition for another 2 h. The absorbance was read by a microplate reader at 450 nm (Bio Tek, Santa Clara, CA, USA, Elx800).

### 3.6. Wound-Healing Assay

Cells were seeded into 6-well plates and cultured in medium supplemented with 10% FBS until reaching approximately 90% confluence. A sterile pipette tip was used to create a linear wound across the monolayer. Detached and floating cells were removed by washing once with PBS. Then, cells were incubated in FBS-free medium (2 mL per well) for 24 h. The wound closure was monitored and imaged at designated time points, and the gap distances were measured to evaluate cell migration capacity.

### 3.7. Colony Formation Assay

Cells were seeded into 6-well plates at a concentration of 500 cell/well and cultured in DMEM supplemented with 10% FBS at 37 °C under 5% CO_2_ condition for 14 days. Then, cells were fixed with 4% polyformaldehyde and stained with 0.1% crystal violet (Aladdin, Shanghai, China, C110703). Visible colonies were counted with ImageJ 1.53 software. The assays were performed in triplicate.

### 3.8. Transwell Migration and Invasion Assay

Cells were seeded into the upper chamber of a Transwell cell culture insert at a density of 3 × 10^4^ cells for both migration and invasion assays in 200 μL of FBS-free medium. The lower chamber was filled with 500 μL of medium containing 10% FBS. After 24 h of incubation, non-migrated or non-invaded cells in the upper chamber were carefully removed using a cotton swab. Cells that had migrated or invaded to the lower side of the membrane were fixed with 4% paraformaldehyde and stained with 0.1% crystal violet (Aladdin, Shanghai, China, C110703). The stained cells were visualized and counted under a microscope. The assays were performed in triplicate.

### 3.9. mCherry-EGFP-LC3 Lentivirus Transfection

GES-1 WT, GES-1 *Hp* F10, GES-1 *Hp* F20, and GES-1 *Hp* F30 cells were seeded into 6-well plates with a density of 2.5 × 10^4^ cells/mL. After cell attachment, the old medium was discarded, and the cells were washed with PBS. Transfection was conducted for 48 h with medium containing a 50 mL titer of 10^8^ TU/mL mCherry-EGFP-LC3 lentivirus (Hanbio Biotechnology Co., Ltd., Shanghai, China) and 8 mg/mL Polybrene. The fluorescence expression of cells was observed to check the efficiency of transfection. When the ratio of fluorescent cells reached approximately 80%, the cells were used for the following experiments.

### 3.10. Apoptosis Assay

The apoptosis level of GES-1 WT, GES-1 *Hp* F1, GES-1 *Hp* F10, GES-1 *Hp* F20, and GES-1 *Hp* F30 cells were analyzed by flow cytometry with an LSR Fortessa Flow Cytometer (BD, San Jose, CA, USA) at 488 nm. Cells were stained using an Annexin V-PE/7-AAD Apoptosis Detection Kit (Vazyme, Nanjing, China, A213-01) according to the manufacturer’s instructions.

### 3.11. RNA Extraction and Real-Time PCR Analysis

Total mRNA was isolated from GES-1 cells and gastric tissues using RNA Isolater Total RNA Extraction Reagent (Vazyme, Nanjing, China; cat. no. R401-01), followed by reverse transcription with 5× All-In-One RT MasterMix (ABM, Richmond, BC, Canada). Quantitative real-time PCR (qPCR) was conducted on a CFX96 Real-Time PCR Detection System (Bio-Rad Laboratories, Inc., Hercules, CA, USA; cat. no. 185-5195) using AceQ qPCR SYBR Green Master Mix or Taqprobe qPCR Master Mix (ABM, Richmond, BC, Canada). All reactions were run in triplicate. Relative transcript levels were normalized to GAPDH mRNA and calculated using the ΔΔCt method. Sequence-specific primers are listed in Supplementary Table S1.

### 3.12. Western Blotting

Cells and tissues were collected and lysed by RIPA Lysis Buffer (Solarbio, R0010) with 1% phenylmethanesulfonyl fluoride (PMSF) (Solarbio, P0100), 1% Phosphatase Inhibitor Cocktail I (MCE, Monmouth Junction, NJ, USA, HY-K0021), and 1% Phosphatase Inhibitor Cocktail II (MCE, HY-K0022). The lysates were centrifuged at 15,000 rpm at 4 °C for 15 min, and the supernatants were collected as protein samples. Protein concentration was determined using a BCA protein assay kit (CWBIO, Beijing, China, CW0014S). Protein samples were diluted with PBS to a unified final concentration, mixed with 6× SDS loading buffer (ROBY, Beijing, China, RBU114-2), and then heated at 99 °C for 10 min. Then, proteins were separated by 10% SDS-PAGE at 80 V for 30 min and then 120 V for 1 h. The separated proteins were electro-transferred onto nitrocellulose membranes (Pall, Port Washington, NY, USA, 66485) at 200 mA for 1 h 45 min. Membranes were then blocked in 10% non-fat milk (OXOID, Waltham, MA, USA, LP0033B) at room temperature for 1 h, followed by overnight incubation at 4 °C with primary antibodies. After washing, membranes were incubated with the appropriate secondary antibodies at room temperature for 1 h.

The following antibodies were purchased: rabbit anti-E-cadherin antibody (Proteintech, Rosemont, IL, USA, 20874-1-AP, 1:5000 dilution), rabbit anti-N-cadherin antibody (Proteintech, Rosemont, IL, USA, 22018-1-AP, 1:2000 dilution), rabbit anti-ZO-1 antibody (Abcam, Cambridge, UK, ab216880, 1:1000 dilution), rabbit anti-Snail antibody (CST, Danvers, MA, USA, 3879T, 1:1000 dilution), rabbit anti-β-actin antibody (HUABIO, R1102-1, 1:1000 dilution), Nod1 (CST, Danvers, MA, USA, USA, 3545S, diluted 1:1000), Mouse monoclonal to p62 (Abcam, UK, ab56416, 1:1000 dilution), anti-LC3B-II antibody (CST, USA 2775, 1:1000 dilution), and HRP-linked goat anti-rabbit IgG antibody (Solarbio, SE134, 1:5000 dilution).

### 3.13. H&E Staining

Stomach tissues were fixed in 4% polyformaldehyde, dehydrated through a serial alcohol gradient, embedded in paraffin, and cut into 4 μm-thick sections. Sections were stained with hematoxylin and eosin (H&E), followed by identification of the pathological status of the sample.

### 3.14. ELISA Analysis

The serum levels of TLR4, TLR5, and TLR9 in gerbils were measured by enzyme linked immunosorbent assay (ELISA) using the Gerbil ELISA kits purchased from Jiangsu Meibiao Biotechnology Co., Ltd. (Yanchen, China) following the manufacturer’s instructions.

### 3.15. Multiplex Assay

The supernatants of wild-type GES-1 cells (GES-1 WT), GES-1 cells exposed to *H. pylori* lysate for 1 generation (Lysate-GES-1 F1), and GES-1 cells with sustained exposure to *H. pylori* lysate for 10 (Lysate-GES-1 F10) and 30 (Lysate-GES-1 F30) consecutive generations were collected, respectively, and used for cytokine determination in Luminex assays. The levels of *H. pylori* infection-induced pro-inflammatory cytokines were measured using technical microspheres with a Luminex X-200 instrument (LUMINEX, Austin, TX, USA).

### 3.16. RNA Sequencing

RNA sequencing was performed by Beijing Novogene Technology Co., LTD. (Beijing, China). Sequencing libraries were generated using NEBNext^®^ Ultra^TM^ RNA Library Prep Kit for Illumina^®^ (NEB, Ipswich, MA, USA). The clustering of the index-coded samples was performed on a cBot Cluster Generation System using TruSeq PE Cluster Kit v3-cBot-HS (Illumia, San Diego, CA, USA). Illumina Casava1.8 software was used for base calling.

### 3.17. Statistical Analysis

Data were analyzed and visualized in GraphPad Prism 8. Independent sample t-test, one-way analysis of variance (ANOVA), and two-way ANOVA were used to determine statistical significance depending on the data type and experimental design. All data represents means ± SD, and statistical significance was defined as *p* < 0.05 (ns represents no significant difference, * *p* < 0.05, ** *p* < 0.01, *** *p* < 0.001, **** *p* < 0.0001).

## 4. Conclusions

In conclusion, this study elucidates a biphasic adaptive process in gastric epithelial cells during *H. pylori* infection. Initially, GES-1 cells undergo stress-induced dysfunction, characterized by suppressed proliferation, impaired motility, blocked autophagic flux, and elevated apoptosis. However, with prolonged bacterial exposure, the cells gradually recover essential functions and develop both phenotypic and immunological tolerance to *H. pylori* (Table 1). Our work provides a cellular-level characterization of the dynamic phenotypic and transcriptional alterations underlying chronic *H. pylori* infection, offering novel molecular insights that underscore the critical importance of early intervention to prevent *H. pylori*-associated gastric pathogenesis.

## Figures and Tables

**Table 1:** Comparison of Acute vs. Chronic *H. pylori* Infection.

Category	Acute Phase	Chronic Phase	Outcome of Chronic *H. pylori* Infection
Morphology	Hummingbird phenotype	Morphology reverts to normal	Cellular adaptation and immune tolerance
CagA Delivery	High cytoplasmic enrichment	CagA signal decreases then rebounds	Impaired toxin clearance
TLR Expression	TLR4/5/9 upregulated	Downregulated	Immune desensitization is established
Cytokine Secretion	IL-1β, IL-8, IL-23, IFN-γ peak	Declines	Attenuated inflammatory response
Cell Proliferation	Decreased	Recovers	Overcoming of stress-induced growth arrest
Migration and Invasion	Reduced	Recovers and then enhanced	Acquisition of motile, invasive phenotype
EMT Markers Expression	Unaffected	E-cadherin/ZO-1 down; Snail up	EMT initiation promotes malignancy
Autophagic Flux	Blocked	Restored	Shift to pro-survival autophagy
Apoptosis Level	Elevated	Declines	Accumulation of damaged cells
Transcriptomic Profile	DNA repair and apoptosis pathways active	NF-κB and pro-migration genes active	Reprogramming to pro-carcinogenic signaling
In Vivo Pathology	No significant changes	Progressive atrophy to dysplasia	Recapitulation of Correa’s cascade
In Vivo TLR Expression	TLR4/9 increased	TLR4/9 decline	Systemic immune tolerance

## Data Availability

The data presented in this study are available on request from the corresponding author.

## Supplementary Materials

The supporting information can be downloaded at: https://www.mdpi.com/article/10.3390/ijms26189016/s1.
