# Supplementary material for: Biphasic Adaptations of Gastric Epithelial Cells in Chronic H. pylori Infection from Stress to Tolerance
Source: Int J Mol Sci. 2025 Sep 16;26(18):9016. doi: 10.3390/ijms26189016 (PMC12469407; doi:10.3390/ijms26189016)
Supplement: Supplementary file 1 [file ijms-26-09016-s001.zip › Supplementary Table S1.pdf]

Supplementary Table S1. The specific primers of TLR and GAPDH

Gene	Sense primer (5' — 3')	Antisense primer (5' — 3')
TLR4	CTCCTGCGTGAGACCAGAAA	CCGTGATAAAACGGCAGCAT
TLR5	CACAGTCACCAAACCAGGGA	GGGCAAAGTCAATTGCCAGG
TLR9	TGCCCAAACTGGAAGTCCTC	TAAGGTTGAGCTCTCGCAGC
GAPDH	CGACAGTCAGCCGCATCTT	CCAATACGACCAAATCCGTTG
